# Biosensors Incorporating Bimetallic Nanoparticles

**DOI:** 10.3390/nano6010005

**Published:** 2015-12-31

**Authors:** John Rick, Meng-Che Tsai, Bing Joe Hwang

**Affiliations:** 1 NanoElectrochemistry Laboratory, Department of Chemical Engineering, National Taiwan University of Science and Technology, Taipei 106, Taiwan; nouveauvous@yahoo.com (J.R.); snowild0326@gmail.com (M.-C.T.); 2 National Synchrotron Radiation Research Center, Hsinchu 300, Taiwan

**Keywords:** biosensor, bimetallic, nanoparticle, electrocatalytic, catalytic

## Abstract

This article presents a review of electrochemical bio-sensing for target analytes based on the use of electrocatalytic bimetallic nanoparticles (NPs), which can improve both the sensitivity and selectivity of biosensors. The review moves quickly from an introduction to the field of bio-sensing, to the importance of biosensors in today’s society, the nature of the electrochemical methods employed and the attendant problems encountered. The role of electrocatalysts is introduced with reference to the three generations of biosensors. The contributions made by previous workers using bimetallic constructs, grouped by target analyte, are then examined in detail; following which, the synthesis and characterization of the catalytic particles is examined prior to a summary of the current state of endeavor. Finally, some perspectives for the future of bimetallic NPs in biosensors are given.

## 1. Introduction

### 1.1. Importance of Biosensors

**Figure 1:**
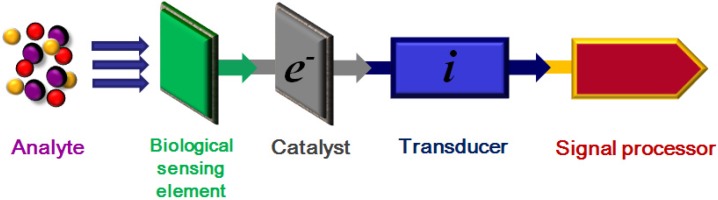
Generalized biosensor schematic showing stages in response to analyte.

A biosensor can be defined as: “a device for the detection of an analyte that couples a bio-recognition element to a signal transducer to generate a measurable electrical signal” [1,2,3], see Figure 1. Biosensors comprise, a sensing element that is ideally able to selectively detect (bind) the analyte of interest when it is presented in a complex biological matrix, a catalyst (if required) to generate a secondary analyte, and a transducer able to generate a response. In Figure 1a signal processor, able to translate the transducer’s response into an intelligible output, is also included. However, such a definition and description of a sensor fails to give any hint of the ongoing rapid evolution of such devices, resulting from the constant progress being made in both materials development and bio-medical research [4,5,6,7,8]. Various nano-materials such as gold nanoparticles (NPs), carbon nanotubes, magnetic NPs and quantum dots, together with graphene based materials [7,8,9,10] are being actively investigated as possible candidate materials for biosensor applications, making them the focus of collaborative interdisciplinary research between the biological sciences, quantitative analytical chemistry disciplines and material science investigators [11,12,13].

The purpose of a biosensor is to provide real-time quantitative information about the chemical composition of the environment in which the sensor is situated. Ideally, such a device should be capable of responding rapidly and continuously, while only minimally perturbing its surrounding matrix. Biosensors have been designed that can detect and measure the multitude of bio-molecules that play crucial and ever expanding roles in diverse areas such as: the bio-monitoring of chemical exposure (human dosimetry) for risk assessment [14,15]; the detection of waterborne pathogens (review article [16]); food safety [17,18,19,20]; diagnostics and physiological monitoring [8,21,22,23,24,25,26,27,28,29]; industrial processes [30,31,32]; environmental monitoring [21,33,34,35,36,37,38] and more recently in security applications, e.g., the detection of biological warfare agents [7,26,39,40,41].

### 1.2. Type of Biosensors—Electrochemical Methods

Ideally, an electrochemical biosensor should unite the sensitivity of an electro-analytical method with the inherent bio-selective capability of a “biological recognition” component. The “biological recognition component” in the sensor, which may be natural or synthetic, recognizes and binds its target analyte, resulting ultimately in the generation of an electrical signal that is able to induce a response, able to be monitored, in a transducer, that is proportional to the target analyte’s concentration.

The foregoing description gives little indication of the difficulties involved in converting information related to the bio-analyte, such as its concentration in any given matrix, into to a signal. Thus the realization of accurate bio-sensing for many targets remains problematic, due to both the sensing environment and the difficulty of connecting an electronic device directly to a biological sensing element [3].

To address these challenges a variety of sensing approaches have been explored, some of which have led to new and innovative devices. Electrochemical methods, because of their simplicity, low power requirements and the ease with which they can be miniaturized, are particularly suitable for development in portable sensing applications [42].

Electrochemical measurements are based on detection or transport of charge across an electrode. Chemical species, such as molecular ions, are referred to as electro-active species if they can either be oxidized or reduced at an electrode’s surface through the movement of electrons [15].

**Figure 2:**
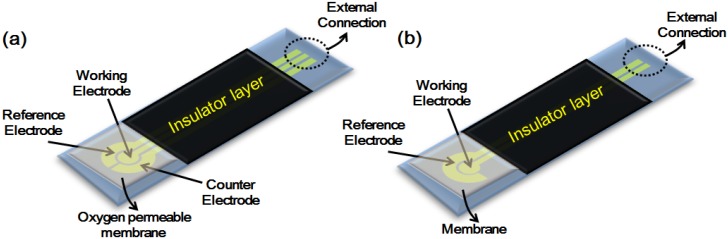
Showing (**a**) amperometric and (**b**) potentiometric electrode configurations.

In this review will give an outline of the two major electrochemistry techniques traditionally employed. The choice of which approach to use depends on the analyte, matrix and sensitivity/selectivity requirements; the first to be considered, namely, amperometry, schematically shown in Figure 2a, employs a detector that measures current when an electro-active solute contacts a working electrode held at a fixed potential with respect to a reference electrode. The second commonly encountered technique potentiometry, schematically shown in Figure 2b, measures the potential at the working electrode, with respect to the reference electrode, in an electrochemical cell when zero or no significant current flows [43].

Amperometry is the term indicating the application of any electrochemical technique in which a current is measured as a function of an independent variable such as time or electrode potential; thus, amperometric sensors are ideally suited for the detection of electro-active species involved in a chemical or biological recognition process [44]. For example, in the example above (Figure 2a) the detected species is envisaged to be O_2_, generated for instance by the reaction of glucose oxidase with its target analyte (glucose). The signal to be measured is generated by a working electrode whose potential is maintained at a constant value (relative to that of a reference electrode) while simultaneously monitoring the current that results from an electro-active solute contacting the working electrode, which is a function of the concentration of the analyte changing over time. An applied potential drives the electron transfer reaction that involves the electro-active species in the solute, thus giving rise to a current representing the rate of the measured reaction in the recognition event, and as such is therefore proportional to the concentration of the target analyte. However, the method suffers from limitations in precision, in that the measured current depends on several other variables that are not always easily controlled. For example, if the working electrode’s applied potential is great enough to reduce the analyte, then the analyte’s concentration in close proximity to the working electrode will be reduced, thus requiring more of the analyte to diffuse into the solution in the region of the working electrode to reestablish the concentration. If the applied potential at the working electrode is sufficient (*i.e.*, an overpotential), then the analyte concentration, in the vicinity of the working electrode, will be entirely dependent on the rate of diffusion. In such a case, the current is said to be *diffusion limited*. As analyte reduction proceeds at the working electrode, its concentration throughout the whole solution will gradually decrease; the rate of this change is dependent upon on the size of the working electrode relative to the total solution volume.

In a potentiometric sensor (Figure 2b), analytical information ultimately results from a process that converts the initial recognition “event”, into a measurable signal, which must be proportional (normally in a logarithmic manner) to the concentration (or the activity) of the species created or consumed in the recognition event. Potentiometric sensors passively measure the potential of a solution between two ion selective electrodes (in the case illustrated: a reference electrode and a Pt electrode made selective with a membrane), and thereby only minimally perturb the solution in the process of obtaining the signal representative of the analytes concentration. The recognition process can be made more selective, with respect to a given target species, by placing a permselective ion-conductive membrane at the tip of the electrode. The most common potentiometric electrode is the glass-membrane electrode used in a pH meter. Potentiometric sensors, being passive devices, measure a response under conditions of essentially zero current, and in doing so offer high selectivity and simplicity at low cost. They are, however, often less sensitive than amperometric sensors designed for the same target species [45].

Readers seeking a more comprehensive introduction to electrochemical sensor principles and architectures that also includes additional techniques such as conductometric devices, cyclic voltammetry (CV), chronoamperometry, chronopotentiometry, electrochemical impedance spectroscopy, the use of field-effect transistors, nanowires, and electrochemical surface-plasmon resonance (SPR) are referred to a comprehensive review paper by Grieshaber *et al.* [46].

### 1.3. Central Issues Related to Biosensors

Specific molecular recognition of a target analyte is a fundamental prerequisite for the operation of a biosensor. Such recognition is based on the affinity between complementary structures; including enzymes and substrates, antibodies and antigens and hormones/signalling molecules and receptors, to generate concentration-proportional signals. A biosensor’s selectivity and specificity are thus highly dependent on the biological recognition systems that are interfaced, through an electrocatalyst, with a suitable transducer. The analysis of bio-analytes remains problematic, due to both the inherently labile nature of the species being detected, which are often present at low concentrations, and also due to the analytical matrix that will frequently contain unknown concentrations of potentially interfering species [42]. At the time of writing there is, with regard to the published literature, a shortage of data regarding issues such as long term material stability, sensor integrity and the reproducibility of results. Such issues, although possibly of less immediate interest to researchers, nevertheless have a significant longer-term impact on the ability of the technology to reach the market place.

Analysis, especially quantitative analysis, of such analytes has traditionally required skilled technicians to use complicated separation techniques, often in conjunction with sophisticated instrumentation and highly labile recognition molecules, such as antibodies and enzymes. Such limitations render these methods unsuitable for point-of-use application, while approaches relying on the incorporation of such species into sensing devices brings with it related issues of stability and storage. Ideally a biosensor should have a fast response time (encompassing at least 95% of the total response), a stable and easily detected end-point, a high signal to noise ratio, a high selectivity, and a broad linear-response range for the analyte of interest.

### 1.4. The Role of Electrocatalysts in Biosensors

Biosensors have been traditionally fabricated using a variety of sensing, or recognition, elements, including enzymes, antibodies, microbes, receptors, cells, membranes, tissues, organisms, organelles, nucleic acids and organic molecules. Such sensors can be tailored, using the natural affinity of the sensing element for its target analyte to give a high degree of specific recognition, such as is commonly seen with e.g., monoclonal antibodies [43].

Several methods are available to translate the signal generated from such interactions into a quantitative representation of the concentration of the target analyte, such methods include: optical methods, e.g., fiber optic (optrode), SPR, fiber optic; calorimetric methods, e.g., heat conduction, isothermal, isoperibol; and acoustic methods e.g., surface acoustic wave, and piezocrystal microbalance [3].

In contrast to sensors in which there is an interaction between the biological element and the analyte that leads to a chemical change in which the concentration of one of the substrates or products changes, catalytic sensors employ an electrocatalyst to either assist in transferring electrons between the electrode and reactants, and/or facilitate a chemical transformation. While the electrocatalyst increases (or decreases) the rate of an electrochemical reaction it is itself not consumed in the process. The overall result of electrocatalyst/substrate binding is to convert the primary substrate into an auxiliary substrate which becomes the species to be quantified using the electrochemical transduction techniques previously referred to [3].

Electrocatalysts either function at electrode surfaces, or they may form the electrode surface itself. An electrocatalyst can be heterogeneous, where it is present as a separate phase, such as a platinum surface or NP; or alternatively, it may be homogeneous, *i.e.*, distributed throughout the reaction medium on a molecular scale, e.g., a coordination complex or enzyme. In this review only heterogeneous bimetallic electrocatalysts will be considered.

**Figure 3:**
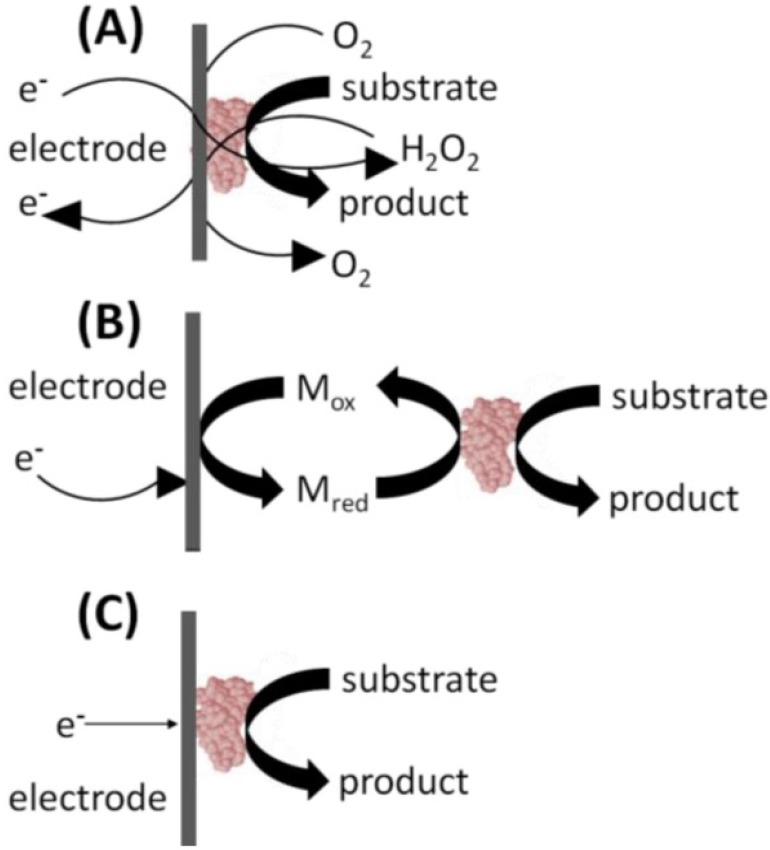
The three generations of biosensors showing transition from: (**a**) enzyme dependency to (**b**) mediator use and (**c**) finally to the use of a catalytic electrode.

Biosensor design progress is reflected in the so-called three “generations” of biosensors [42], see Figure 3. The first generation of biosensors dates back to the first glucose biosensor developed by Clark and Lyons. First generation biosensors for glucose sensing used an oxidase enzyme, *i.e.*, glucose oxidase (GO*_x_*) in conjunction with a dialysis membrane immobilized on the surface of a platinum electrode (Figure 3a). GO*_x_* is a selective catalyst whose action gives rise to the production of hydrogen peroxide (H_2_O_2_) that can be measured at a platinum electrode. Today most commercial bench-top amperometric biosensors rely on reactions catalyzed by oxidase enzymes, followed by the detection of H_2_O_2_ on Pt electrodes. The problem with this design is the loss in selectivity that occurs between the bio-recognition event and the amperometric H_2_O_2_ detection, while additionally the highly oxidizing potential (700 mV *versus* Ag/AgCl) necessary for H_2_O_2_ oxidation may result in unacceptable levels of interference from the oxidation of other electro-active species in complex analytical matrices.

Second-generation biosensors, which have been commercialized, mostly in single-use testing format, use an artificial electron mediator to generate an improved sensing response (Figure 3b). Thus, the concentration of the analyte is measured not by the rate of disappearance of substrate, or appearance of product; but by the rate of electron flow from the analyte to the surface of an electrode. Ferrocene, quinones, quinoidlike dyes, and organic conducting salts, have been used as mediators. Eliminating the O_2_ dependence of the first-generation method helped to facilitate control of the enzymatic reaction and thereby improve sensor performance.

Third generation sensors originally attempted to co-immobilize the enzyme and mediator on an electrode’s surface, thus making the bio-recognition component an integral part of the electrode transducer. This approach has led in turn to the direct electrical contact of enzyme and electrode, and more recently to the reaction being promoted by the catalytic structure itself giving rise to the response with no product, or mediator, diffusion being directly involved, see Figure 3c. Such devices eliminate the need to accurately relate the transfer of electrons between the electrode and enzyme. Work aimed at the commercialization of such devices is ongoing.

### 1.5. Scope

While there is an abundance of literature focused on monometallic catalysis and their application to all manner of conversion reactions, the volume of literature regarding the use of bimetallic constructs is only now starting to expand. The introduction of a second metal into a catalytic structure potentially affords opportunities for both catalytic fine-tuning, resulting from bimetallic complimentarity and synergisms; together with cost-savings, resulting from the potential to reduce the amount of the more expensive metals, e.g., Pt. This review will cover the fundamentals and bio-applications of biosensors that employ bimetallic constructs. The two classes of bimetallic electrochemical biosensors, namely bio-catalytic devices and affinity sensors, will be discussed to provide an accessible introduction to the field of electrochemical biosensors. This review is arranged in the order of target species. A table is presented for each target analyte; within this table the bimetallic constructs that have been used are arranged in reverse chronological order.

While sensors for the more commercially important analytes, e.g., H_2_O_2_ and glucose tend to attract the most interest there is a growing body of work focused on diverse new, interesting and important bio-analytes. The sensing targets in this review are: H_2_O_2_, glucose, uric acid (UA), cholesterol, carcinoembryonic antigen (CEA), long non-coding RNA, carbohydrate antigen 19-9, cancer cells (human leukemia CCRF-CEM), dopamine, cardiac troponin I, human tissue polypeptide antigen (hTPA), cancer antigen 125 (CA-125), glutamate, lead, mercury, zearalenone (ZEA), organophosphates and 2-butanone.

## 2. Biosensors—Grouped by Target Analyte

### 2.1. Hydrogen Peroxide Sensing: See also Section 2.3, Combined H_2_O_2_ and Glucose Sensing

Unambiguously sensing and quantifying H_2_O_2_ is becoming ever more become crucial to a wide range of healthcare, industrial, and now more recently, domestic and international anti-terrorism applications. Some of the diverse applications in which H_2_O_2_ sensing finds application are: in paper production for bulk-scale bleaching, in all manner of aseptic packaging, in the rapidly expanding field of bio-imaging, and in the clinical setting for the routine determination of clinically significant analytes such as glucose and metabolites e.g. UA. Despite the industrial, scientific and clinical importance of H_2_O_2_, its direct detection, from e.g. the selective enzymatic breakdown of glucose or urea, remains problematic, due to the fact it shows no UV absorbance or fluorescence. Thus, the development of a robust and sensitive approach to H_2_O_2_ detection without interference from commonly coexisting electroactive agents remains an ongoing quest. In contrast to the focus in this review upon sensors solely using bimetallic constructs, a recent general review of H_2_O_2_ sensing giving interested readers an overview of the subject area has recently appeared [47]. Table 1 below shows relevant bimetallic constructs used for H_2_O_2_ sensing together with resulting detection limits and sensitivities.

In terms of the bimetallic constructs used the approaches mentioned in the table can be divided into several groups. The four papers published between 2009 and 2011 all employ an active catalytic metal supported on Au. While the commonality in the use of gold is interesting—possibly suggesting a convergence of approach, the diversity of the second metal may also be indicative of a fundamental lack of mechanistic understanding. These papers, and the bimetallic constructs they used are: Manivannan and Ramaraj [58] (Au/Ag); Che *et al.* [57] (Au/Pt); Tsai *et al.* [56] (Au/Ag); and Yu *et al.* [55] (Au/Pt) who used approaches based on supports comprising, core-shells on silica/sol-gel networks, DNA-l-cysteine-polypyrrole, nano-films formed on modified glassy carbon electrodes (GCEs), and alloyed NPs, respectively. Manivannan and Ramaraj [58] noted that a modified electrode made with an Au/Ag bimetallic construct (ratio 73:27) showed a better synergistic electrocatalytic effect towards the reduction of hydrogen peroxide compared to similarly constructed mono-metal Au and Ag electrodes. An Au/Pt bimetallic synergism was commented on by both Yu *et al.* [55] and Che *et al.* [57]. Yu *et al.* [55] noted that the fabricated construct: “facilitated electron transfer and the intrinsic catalytic activity of Au/Pt NPs provide a facile way to construct a third-generation H_2_O_2_ biosensor with a high sensitivity, low detection limit, quick response time, and excellent selectivity”. Che *et al.* [57] commented on the synergism between Au and Pt as contributing to an improvement in the analytical performance when compared to an electrode modified with Au NPs or Pt NPs alone. Interestingly the seven most recent papers, all third generation sensors using non-enzymatic, mediator-free approaches—namely, those by Li *et al.* [48], Kung *et al.* [49], Hwang *et al.* [50], Lu *et al.* [51], Janyasupab *et al.* [52] and Chen *et al.* [53] have shown a convergence in approach with respect to both the nature of the constructs employed, *i.e.*, all containing Pt, and using carbon, in various forms, as a support material.

**Table 1:** Metals and constructs used for H_2_O_2_ sensing: see also Section 2.3, combined H_2_O_2_ and glucose sensing.

Metals	Construct/Support	Year	Authors	Detection Limits/Sensitivities
Au/Pt	Triple-layered core-shell NPs on GO with both metals exposed in outer layer.	2015	Li *et al.* [48]	Linear range 0.05 mM to 17.5 mM (LOD 0.02 mM) applied potential +0.5 V and linear range 0.5 mM to 110 mM. (LOD 0.25 mM) applied potential −0.3 V.
Pt/Ru	3-D graphene foam supported bimetallic nanocatalysts.	2014	Kung *et al.* [49]	Linear range 0 to 0.2 mM. Sensitivity 1023.1 mA·mM^−1^·cm^−2^. LOD 0.04 mM.
Pt/Ir	Carbon supported NPs.	2014	Hwang *et al.* [50]	Linear ranges 0–1 mM and 2–10 mM, show sensitivities of 18.12 and 7.97 µA·mM^−1^·cm^−2^ respectively.
Pt/Au	Graphene/CNT hybrid.	2013	Lu *et al.* [51]	Linear detection from 2.0 to 8561 μM. Sensitivity 313.4 μA·mM^−1^·cm^−2^.
Pt–M (M = Cu, Ni, Pd, and Rh)	PtM NPs synthesized using oleylamine assisted modified template-free self-assembly approach giving bicontinous network among clusters on PtM/C/Nafion modified GCE.	2013	Janyasupab *et al.* [52]	PtCu/C, PtNi/C, PtPd/C, PtRh/C. Linear ranges (upper limits, mM) 4.0, 2.0, 3.0, and 2.0. Sensitivities (µA·mM^−^^1^·cm^−^^2^) 69.4, 208.5, 239.9, and 839.9. LOD 12.2, 31.5, 114.0, and 34.8. All measured Ag/AgCl (V) +0.3.
PtM (M = Pd, Ir)	CNT.	2012	Chen *et al.* [53]	Linear range (Pt/Pd) 2.5–125 µM. Sensitivity 414.8 µA·mM^−^^1^·cm^−^^2^. LOD 1.2 µM.
Rh/Pd	NPs supported on GCE/ITO.	2011	Rajkumar *et al.* [54]	Linear ranges 10–460 µM (lab sample) sensitivity 0.3160 µA·µM^−1^ and 10–340 µM (real sample) sensitivity 0.0560 µA·µM^−1^.
Au/Pt	Alloyed NPs.	2011	Yu *et al.* [55]	Linear ranges: 0.1–12 µm, sensitivity 3.36–1.02 nA·mm^−1^ and 5 mm to 0.25 mm, sensitivity 20.09–1.93 nA·mm^−1^. LOD 60 nm.
Au/Ag	Nano Au–Ag film formed on modified GCE.	2010	Tsai *et al.* [56]	Linear range 1–250 µM in lab samples, and 1 × 10^−3^–2 × 10^−2^ M in real samples.
Au/Pt	DNA-L-Cysteine- Polypyrrole.	2009	Che *et al.* [57]	Linear CV and chronoamperometry responses between 4.9 μM and 4.8 mM. LOD 1.3 μM.
Au/Ag	Core-shells on Silica/Sol-Gel network.	2009	Manivannan and Ramaraj. [58]	Linear range 10 μM to 70 μM H_2_O_2_. LOD 1 μM.
Cu/Fe	Bimetallic porphyrin film.	2003	Vago *et al.* [59]	N/A
Ru/Rh	Au supported.	2002	Janasek *et al.* [60]	Linear ranges 1–1000 μM (potential −100 mV) and 2–500 μM (potential +250 mV) *vs.* Ag/AgCl/0.4 M KCl.

In an interesting earlier approach to sensor formation Janasek *et al.* [60] used radio frequency sputtering to form ruthenium/rhodium modified gold electrodes for the low potential detection of H_2_O_2_. The Ruthenium layers were radio frequency magnetron sputtered and the rhodium layers were added by vacuum evaporation. H_2_O_2_ was able to be detected using cathodic reduction at potentials lower than +170 mV, or by anodic oxidation at higher potentials. In contrast Vago *et al.* [59] were inspired by a “nature analogy” to create a CuFe bimetallic porphyrin film able to act as a first generation biosensor: they noted that a unique Fe/Cu interaction is observed in biological systems, in which cytochrome c oxidase, a membrane-bound metalloenzyme, efficiently catalyses the reduction of oxygen to water. Interestingly the authors commented that the use of polyCu protoporphyrin IX deposited on polyFe protoporphyrin IX allows for the preservation of catalytic properties over a wide pH range.

### 2.2. Glucose Sensing: See Also Section 2.3, Combined H_2_O_2_ and Glucose Sensing

The seemingly inexorable rise in the number of obese people in the developed world is mirrored by a parallel increase in diabetes (both diagnosed and undiagnosed) and its related pathological consequences, e.g., blindness, circulation problems leading to amputation, kidney and other organ failure [61,62,63,64]. This morbidity and its economic impact is the prime reason for the research focus on the measurement of blood glucose levels, especially using cheap disposable amperometric sensors in the home-care setting for the monitoring of blood sugar levels, at regular intervals, in diabetic patients [4]. Readers seeking an introduction to electrochemical glucose sensors are referred to two excellent review articles published by Wang in 2008 and 2001 [25,65]. A further contemporary review specifically focused solely on electrochemical glucose sensing, without specific focus on bimetallic catalysts, complimentary to the present article is offered by Chen *et al.* [66]. Several approaches to the electrocatalysis of glucose have employed one-dimensional materials, e.g., nanowires and nanotubes. A review of these materials, used as supports for catalytic Pt/Pd, for non-enzymatic glucose sensing, in which their respective performances are compared to commercial Pt/C and Pt black catalysts is offered by Li *et al.* [67] Table 2 below shows bimetallic constructs that have been used for glucose sensing.

**Table 2:** Metals and constructs used for glucose sensing.

Metals	Construct/Support	Year	Authors	Detection Limits/Sensitivities
Au/Ag	Bimetallic constructs formed as heterogeneous nanorods with peroxidase-like activity.	2015	Han *et al.* [68]	Linear range 0.05–20 mM. LOD 25 μM
Au/Cu	Construct made as nanowires.	2015	Kim *et al.* [69]	Suggested use of the NWs is for monitoring glucose levels in single cells or as microsystem electrocatalysis.
Cu/Ag	Cu/Ag nanocomposite with low Ag content and rough surface and algal-like microstructure.	2015	Li *et al.* [70]	CV, chronoamperometry and EIS show sensitivity up to 7745.7 μA·mM^−1^·cm^−2^, LOD 0.08 μM.
Cu/Au	Gold-incorporated copper (Cu/Au) nanostructures.	2015	Tee *et al.* [71]	Linear detection range 0 to 5.5 mM. Sensitivity 1.656 mA·mM^−^^1^·cm^−^^2^. LOD 2 μM.
Pd/Cu	NP decorated three-dimensional graphene hydrogel.	2014	Yuan *et al.* [72]	Linear range to 18 mM, with applied potential −0.4 V reproducible sensitivity 48 µA (mg·mM^−^^1^).
Pt/Au	DNA-templated synthesis of bimetallic NP/graphene nanocomposites.	2014	Leng *et al.* [73]	Linear range 1.0 to 1800 μM. LOD 0.3 μM.
Pt/Pd	Highly dispersed Pt/Pd NPs/RGO composite formed in one-pot synthesis.	2014	Li *et al.* [74]	Linear range 0.1 to 22 mM at 0 V
Pt/Pd	Graphene nanosheets modified with Pt/Pd nanocubes built on electrode.	2014	Chen *et al.* [75]	Linear to 24.5 mM applied potential +0.25 V. Sensitivity 1.4 μA·cm^−2^·M^−1^.
Ag/Ni	Bimetallic alloys deposited on GCE.	2013	Miao *et al.* [76]	Two linear ranges, 1 to 9.89 μM (sensitivity 6.48 nA/μM), and 19.68 to 106.89 μM (sensitivity 2.88 nA/mM). LOD 0.49 μM. S/N ratio 3.
Au/Pt	Construct made as nanowires.	2012	Mayorga-Martinez *et al.* [77]	Linear response range up to 140 µm with 8557 Ω·mM^−1^.
Pt/Pd	NP on multi-walled CNTs.	2012	Chen *et al.* [78]	Linear range (0.062–14.07 mM). LOD 0.031 mM. Sensitivity 112 µA·mM^−^^1^·cm^−^^2^.
Pt/Te	Te microtubes on Pt electrode.	2012	Guascito *et al.* [79]	Linear between 0.1 and 1 mM sensitivity 522.61 μA·cm^−2^·mM^−1^ and between 1 and 29 mM sensitivity 62.45 μA·cm^−2^·mM^−1^. LOD 0.1 mM.
Au/Pt	Ni(II)–pyromellitic acid with bimetallic construct on GCE.	2012	Gholivand & Azadbakht. [80]	Linear response from 100 nM to 100 µM. LOD 55 nM.
Pt-M (M = Ru & Sn)	CNT.	2012	Kwon *et al.* [81]	Linear range (mM), sensitivity (A·mM*^−^*^1^), LOD (mM) (S/N = 3) for Pt-Ru were 1.0–2.5, 18.0, 0.7, respectively. Using Pt-Sn the corresponding figures were 1.00–3.00, 889.0 and 0.3.
Pt/Ni	Graphene/Pt/Ni alloy NP nanocomposites.	2011	Gao *et al.* [82]	Linear response (under physiological condition) to glucose concentrations up to 35mM. Sensitivity 20.42 μA·cm^−2^·mM^−1^ at −0.35 V.
Au/Pd	RGO and Au/Pd (1:1).	2011	Yang *et al.* [83]	Linear range up to 3.5 mM. LOD 6.9 µM. Sensitivity 266.6 µA·mM^−^^1^·cm^−^^2^.
Cu/Au	Bimetallic electrode.	2011	Shi *et al.* [84]	Linear response 200 nM and 10 mM. LOD 50 nM (S/N = 3), Current response 40.8 μA·mM^−^^1^·cm^−^^2^.
Pt/Pd	Mesoporous carbon vesicles.	2011	Bo *et al.* [85]	Linear range 1.5 to 12 mM. LOD 0.12 mM. S/N ratio = 3. Sensitivity of 0.11 μA·mM^−^^1^·cm^−^^2^.
Cu/Ni	Dendritic materials on titanate films.	2010	Tong *et al.* [86]	Linear response from 1.0 × 10^−^^6^ to 5.0 × 10^−^^4^ M. Sensitivity 661.5 μA·mM^−^^1^. LOD 3.5 × 10^−^^7^ M.
Au/Pd	Core shell NPs on GCE.	2010	Chen *et al.* [87]	Linear response from 5 nM–0.5 µM. LOD 1.0 nM (S/N = 3).
PtM (M = Ru, Pd & Au)	CNT-Ionic liquids used to form sensor on GCE.	2009	Xiao *et al.* [88]	Linear response to 15 mM (−0.1 V app. potential). LOD 0.05mM (S/N = 3). Sensitivity 10.7 µA·cm^−^^2^·mM^−^^1^.
Pt/Pb	Ti substrate.	2008	Wang *et al.* [89]	Linear current response to 15 mM. Sensitivity of 10.8 µA·cm^−^^2^·mM^−^^1^.
Cu/Au	Cu highly dispersed on gold.	1997	Casella *et al.* [90]	Linear range 0.6–52 mM. Sensitivity 1.2 mA·mM^−1^·cm^−2^ (at 0.55 V). LOD 0.8 pmol (S/N = 3).

For reasons of stability, handling and storage, the use of an enzyme in a sensor presents an additional level of complexity that needs to be balanced against the additional selectivity it offers. Of the above group, only the papers by Chen *et al.* [78] and Yang *et al.* [83] used an enzymatic approach. Chen *et al.* [78] constructed a biosensor by immobilizing PtPd-MWCNTs in a Nafion film on a GCE. An inner Nafion film coating was used to eliminate common interferents including UA and AA. Electrodeposition was used to form a porous surface structure with a well-ordered three-dimensional enzyme layer. The biosensor had high reproducibility, good storage stability and a satisfactory anti-interference ability even when challenged with its target analyte in an actual serum sample. Yang *et al.* [83] detailed a simple, fast, green and controllable approach to the electrochemical synthesis of a novel nano-composite of electrochemically reduced graphene oxide (ERGO) and Au-Pd NPs, without the aid of any reducing reagent. A biosensor, with acceptable reproducibility, good accuracy and negligible interference from common oxidizable interfering species, was constructed by immobilizing glucose oxidase on the nanocomposites.

As already noted the incorporation of enzymes into various films formed on electrodes, or on various other supports, such as graphene, has attendant problems such as the maintenance of the enzymes structural integrity as well as issues related to accessing its active site. The replacement of inherently labile enzymes with tailored metallic or bimetallic catalysts, tuned to facilitate the direct electrocatalytic oxidation of glucose at a non-enzymatic electrode, obviously obviates the need for such immobilisation techniques. To date many non-enzymatic glucose sensors have been investigated, especially Pt-based amperometric glucose sensors; however, in general the selectivity and sensitivity of such sensors is not adequate for routine point-of-use practical applications. Therefore, the quest to find alternative cheaper materials suitable for use as selective glucose electrocatalysts that compete in terms of activity with Pt is ongoing.

Bo *et al.* [85] used a facile microwave irradiation method to prepare a nanosized Pt/Pd bimetallic alloy NPs on a lamellar structured mesoporous carbon vesicle (MCV) template. A non-enzymatic amperometric sensor of glucose based on the Pt/Pd/MCV modified GCE was developed. Compared with a monometallic Pt/MCV nanocomposite, the Pt/Pd/MCV modified electrode displayed an enhanced current response towards glucose thereby demonstrating the advantage of the bimetallic construct.

Ultrasonic-electrodeposition was used by Xiao *et al.* [88] to make highly dispersed alloyed PtM (M = Ru, Pd and Au) NPs on composite MWNTs–ionic liquid (trihexyltetradecylphosphonium bis(trifluoromethylsulfonyl)imide). Comparison of results obtained with the bimetallic construct fabricated on a GCE with those from a hospital using actual clinical samples showed close agreement. Radiolytic deposition was used by workers led by Kwon [81] to form sensors comprising MWNTs with highly dispersed alloyed PtM (M = Ru and Sn) NPs (PtM@PVP-MWNTs). Electrochemical testing showed that these non-enzymatic sensors, which were able to avoid interference arising from the oxidation of common interfering species, e.g., AA and UA, generated larger currents than those of either a bare GC electrode or a GC electrode modified with MWNTs.

Approaches to sensor formation on GCEs were used by Miao *et al.* [76] (Ag/Ni); Gholivand and Azadbakht [80] (Au/Pt); and Chen *et al.* [87] (Au/Pd). Claims to exploit synergistic effects were made by Miao *et al.* [76] (Ag/Ni), who commented: “Especially, the presence of Ag improves the elecrocatalytical performance of Ni at lower potential, which facilitates amperometric measurements of glucose and shows the potential to develop glucose based sensors”.

In Iran workers led by Gholivand [80] formed a Ni(II)–pyromellitic acid (PMA) film immobilized on the surface of a bimetallic Au–Pt inorganic–organic hybrid nanocomposite carbon nanotube GCE to fabricate an electrochemical sensor for glucose, while a novel nonenzymatic glucose sensor based on flower-shaped (FS) Au@Pd core-shell NPs-ionic liquids (ILs, *i.e.*, trihexyltetradecylphosphonium bis(trifluoromethylsulfonyl) imide, [P(C_6_)_3_C_14_][Tf_2_N]) composite film modified GCE has been reported by Chen *et al.* [87].

Although bimetallic constructs consisting of Pt plus another metal tend to dominate the group, some of the more recent papers have eschewed the use of Pt. Instead catalytic parings featuring Au and Cu, such as those by Kim *et al.* [69] (Au/Cu); Tee *et al.* [71] (Cu/Au); Han *et al.* [68] (Au/Ag); Li *et al.* [70] (Cu/Ag); and Yuan *et al.* [72] (Pd/Cu), have come dominate. Other earlier approaches (1997 to 2011) that did not include the use of Pt, include the papers by Shi *et al.* [84], Tong *et al.* [86], and Casella *et al.* [90], all of whom used Pt-free bimetallic constructs based on copper, namely Cu/Au, Cu/Ni, and Cu/Au respectively. As is common in the currently published sensor literature, the referenced papers give a good account of the sensor construction procedure and subsequent analytical data; however, details of the rationale leading to the choice of metal pairings frequently remains elusive.

The variety of approaches explored by the above researchers may be indicative of the problematic nature of non-enzymatic direct oxidation of glucose, arguably resulting from a lack of fundamental understanding of the electronic processes involved at the atomic level. The use of metallic electrodes, comprising (either singly, or in combination) Au, Cu, Fe, Ni, and Pt presents two main problems: the first problem is the low sensitivity that results from the “sluggish” kinetics found with glucose electro-oxidation, the second problem is the poor selectivity that originates in the blockage of the electroactive surface by chemisorbed intermediates. In “real” samples such species are commonly endogenous species, e.g., ascorbic acid and uric acid, which can also be oxidized in the potential range used for glucose oxidation.

### 2.3. Combined Peroxide/Glucose Sensing

Each of the above sensors (Table 2) was initially designed to sense H_2_O_2_ (non-enzymatically) using a different bimetallic construct, with the glucose sensing capacity being conferred later in each case by the incorporation (immobilisation) of GO*_x_* in the sensing construct. Table 3 gives details of bimetallic constructs that have been used for the joint sensing of H_2_O_2_ and glucose. Note that in each case detection limits and sensitivities are given for both analytes.

The electrocatalytic behavior of the amperometric non-enzymatic bimetallic combined peroxide/glucose sensor based on a Pt/Pd construct made by Niu *et al.* [93] showed that Pt/PdBNC significantly enhances the electrochemical reduction of H_2_O_2_ in neutral media, exhibiting a preferable electrocatalytic performance compared to Pt and Pd monometallic nanoclusters. Liu *et al.* [94] used nanoporous copper obtained by dealloying CuAl alloy as both a three-dimensional template and as a reducing agent for the fabrication of nanoporous Pd/Cu alloy with hollow ligaments by a simple galvanic replacement reaction with aqueous H_2_PdCl_4_. The nanotubular mesoporous Pd/Cu alloy structure exhibited an improved electrocatalytic activity towards the oxidation of formic acid and H_2_O_2_ compared with nanoporous monometallic Pd. When coupled with GO*_x_*, the enzyme modified electrode could sensitively detect glucose with minimal interference from AA and UA (0.2 mM each analyte), as a result of using a Nafion membrane to act as an effective permselective barrier.

**Table 3:** Metals and constructs used for sensors designed jointly for H_2_O_2_ and glucose.

Metals	Construct/Support	Year	Authors	Detection Limits/Sensitivities
Pd/Pt	Bimetallic NPs embedded in RGO.	2015	Hossain and Park [91]	H_2_O_2_ linear response range 0.5 to 8 mM, sensitivity 437.06 µA·mM^−1^·cm^−2^.
				Glucose (with GO) linear range 0.5 mM to 8 mM; sensitivity 27.48 µA·mM^−1^·cm^−2^.
AuM (M = Pd, Rh, Pt)	Monodispersed AuM (M = Pd, Rh, Pt) bimetallic nanocrystals synthesized in oleylamine solvent.	2015	Han *et al.* [92]	H_2_O_2_ detection limit 8.4 µM. Sensitivity 195.3 µA·mM^−1^·cm^−2^ at 0.25 V *vs.* SCE.
				Glucose linear relationship from 0.5 to 10 mM sensitivity 152.13 µA·mM^−1^·cm^−2^.
Pt/Pd	Snowflake-like bimetallic nanoclusters on screen-printed (SP) Au film electrode.	2012	Niu *et al.* [93]	H_2_O_2_ linear response from 0.005 to 6 mM. Sensitivity 804 µA·mM^−1^·cm^−2^.
				Glucose (with GO) linear range 0–16 mM. LOD 10 mM.
Pd/Cu	Nanoporous PdCu alloy electrode.	2011	Liu *et al.* [94]	H_2_O_2_ linear response 8.0 mM. LOD 0.1 µM.
				Glucose linear in range 0.5–20 mM. LOD 0.1 µM.
Pt/Au	NP-decorated titania nanotube array.	2008	Kang *et al.* [95]	H_2_O_2_ linear response 10 and 80 μM. LOD 10 μM. Response slope 2.92 μA·mM^−1^.
				Glucose (with GO*_x_*) linear response 0 to 1.8 mM. LOD 0.1 mM. Sensitivity 0.08366 μA·mM^−1^.

### 2.4. Uric Acid Sensing

UA is final product of purine nucleotide catabolism. It can present a clinical problem for humans, due to its limited solubility, especially in acidic environments. In human blood plasma, the reference ranges for UA are typically 200–430 µmol/L for men and 140–360 µmol/L for women. Persistent concentrations of UA beyond these levels, *i.e.*, saturating concentrations, can give rise to urate deposits in extracellular fluids, especially the synovial fluids of the joints, in the form of monosodium urate crystals (MSU). The presence of MSU is a clinical determinant of gout a condition in which patients commonly present with inflammatory arthritis accompanied by excruciating pain [96,97]. UA is a diprotic acid with pKa_1_ = 5.4 and pKa_2_ = 10.3. Thus in high pH (alkaline) environments [96,98,99,100,101,102,103,104,105,106,107,108] it forms the doubly charged urate ion; however, at biological pH values it forms the singly charged urate ion as its pKa_2_ value, *i.e.*, second ionization value, is so weak doubly charged urate salts tend to hydrolyze back to the singly charged state at near-neutral pH values [109]. Thus, UA can be treated as a simple monoprotic acid in this pH range due to its limited solubility; however, in urine whose pH value is ~5.7, the potential total contribution must be considered as the totality of urate and UA. Table 4 shows the bimetallic constructs that have been used for UA detection.

**Table 4:** Metals and constructs used for sensors designed to sense uric acid.

Metals	Construct/Support	Year	Authors	Detection limits/sensitivities.
Pd/Pt	Reduced RGO.	2013	Yan *et al.* [110]	Linear detection range 4–400 µM. LOD 0.10 µM.
Pt/Au	Multiwall CNTs.	2007	Yogeswaran *et al.* [111]	Sensitivity (µA·mM^−1^) 153.4 (individual analyte) and 500.4 in mixture of AA, UA and EP. CV used for determination.

Clinical UA biosensors have long relied on enzymatic approaches that are not within the scope of this review. Readers seeking a review of enzymatic UA biosensors are referred to a review by Erden and Kilic [112].

Interestingly both Yogeswaran *et al.* [111] and Yan *et al.* [110] developed sensors for multi-analyte determinations, *i.e.*, AA, epinephrine, and UA; and AA, dopamine and UA respectively. Yogeswaran *et al.* [111], developed a composite material, based on multi-walled CNTs, with promising catalytic activity towards the oxidation of mixture of biochemical compounds, thereby allowing the simultaneous measurement using CV and differential pulse voltammetry of ascorbate anion, epinephrine and urate anion in aqueous buffer solution (pH 6.75). Well-separated voltammetric peaks were obtained for ascorbate, epinephrine and urate anions with peak separations of 0.222 and 0.131 V. While Yan *et al.* [110] synthesized Pd–Pt bimetallic NPs anchored on functionalized RGO in a one-step *in situ* reduction process, in which Pt and Pd ions were first attached to poly(diallyldimethylammonium chloride) functionalized graphene oxide sheets. The encased metal ions and GO were simultaneously reduced by EG. An electrochemical sensor based on the graphene nanocomposites was fabricated and was able to simultaneously detect using differential pulse voltammetry measurements AA, dopamine and UA in a ternary mixture. The practical utility of the sensor was demonstrated by the quantitative determination of the target analytes in human urine and blood serum samples.

### 2.5. Cholesterol

The medical literature makes reference to a host of cardiovascular risk factors, as discussed in numerous reviews [113,114]. Despite all the competing risk factors such as diet, inflammation, infection, lifestyle, *etc.*, the presence of excess cholesterol, especially when it is bound to a low-density lipoprotein carrier, continues to be considered a key risk factor for cardiovascular disease [115,116,117].

Biosensors for the electrochemical determination of cholesterol typically rely on either the consumption of oxygen or the production of H_2_O_2_ by immobilized cholesterol oxidase. Thus, the use of functionally tailored bimetallic constructs, comprising alloyed metal particles, able to address problems associated with, e.g., interference and overvoltage effects, offers a route to efficiently catalyzing the oxidation and reduction of H_2_O_2_.

Both Pt/Pd and Au/Pt NPs [118,119] and TiO_2_/graphene supported Pt/Pd nanocomposites have been used for cholesterol sensing, see Table 5.

Cao *et al.* [119] in a paper entitled: “Electrochemistry of cholesterol biosensor based on a novel Pt–Pd bimetallic NP decorated graphene catalyst” demonstrated a new electrochemical biosensor with enhanced sensitivity for cholesterol detection by using a platinum–palladium–chitosan–graphene hybrid nanocomposite (Pt/Pd–CS–GS) functionalized GCE. The authors commented that the presence of the Pt/Pd–CS–GS nanocomposites not only accelerated direct electron transfer from the redox enzyme to the electrode’s surface, but also enhanced the immobilization cholesterol oxidase (ChO*_x_*). The resulting biosensor had a high specificity to cholesterol with the near-complete elimination of interference from UA, AA, and glucose. The same workers led by Cao also developed an integrated sensing system for detection of cholesterol based on TiO_2_–graphene–Pt–Pd hybrid nanocomposites (TGPHs).

**Table 5:** Metals and constructs used for sensors designed for cholesterol sensing.

Metals	Construct/Support	Year	Authors	Detection Limits/Sensitivities
Pt/Pd	NP decorated chitosan–graphene hybrid nanocomposite incorporating ChO*_x_*.	2013	Cao *et al.* [119]	Linear range (using CV/amperometric detection) 2.2 × 10^−6^ to 5.2 × 10^−4^ M, LOD 0.75 mM. Response time <7 s.
Pt/Pd	TiO_2_–graphene on GCE with ChO*_x_*.	2013	Cao *et al.* [120]	Linear range 5.0 × 10^−8^–5.9 × 10^−4^ M. LOD 0.017 µM. Response time <7 s
Au/Pt	ChOx/AuPt–Ch–IL/GCE.	2011	Safavi *et al.* [118]	Linear ranges (i) 0.05–6.2 mM and (ii) 6.2–11.2 mM. Sensitivity 90.7 µA·mM^−^^1^·cm^−^^2^. LOD 10 µM.

The TGPHs increased the sensing surface area while simultaneously improving the electronic transmission rate [120]. High loading amounts of AuNPs and ChO*_x_* were successively self-assembled to TGPHs. The fabricated biosensor was tested using real food samples such as egg, meat, margarine and fish oil, and showed that it had the potential to be used as a facile cholesterol detection tool in food and quality control applications.

Safavi *et al.* [118] used electrodeposition method to form AuPt alloyed NPs on GCEs modified with a mixture of an IL and chitosan (Ch) (Au/Pt–Ch–IL/GCE). Au/Pt–Ch–IL/GCE was found to electrocatalyse the reduction of H_2_O_2_ making it suitable for the preparation of biosensors, while cross-linking between ChO*_x_* chitosam was used to immobilize the enzyme on the electrode’s surface. The addition of potentially interfering species such as AA and glucose (both 1 mM) elicited no change in the cholesterol response current.

### 2.6. Carcinoembryonic Antigen (CEA)

Carcinoembryonic antigen CEA is a glycoprotein involved in cell adhesion that is normally only produced in gastrointestinal tissue during fetal development *in utero* with production almost stopping prior to birth. As such it should not be present in the blood of healthy adults. CEA is reported to be a tumor marker in colorectal cancer [121] and also possibly, ovarian [122,123] lung cancer [124] and breast cancer [125] where by interfering with cellular adhesion processes it may be crucial to metastatic dissemination. Thus, identifying defined tumor markers allows for the better selection of treatment options related to envisaged prognostic outcomes [124].

CEA, being normally only fetally expressed, should only appear in the blood of healthy adults in very low concentrations (approximately 2.5 ng/mL). However, its presence in amounts significantly greater than this in people suffering from certain types of cancer, especially colorectal carcinoma, means that it can be used as a tumor marker in clinical tests [122,124,125,126]. The approaches published using bimetallic constructs for the detection of CEA are shown in Table 6.

**Table 6:** Metals and constructs used for sensors designed for carcinoembryonic antigen (CEA) sensing.

Metals	Construct/Support	Year	Authors	Detection Limits/Sensitivities
Au/Pt	Bimetallic nanochains.	2013	Cao *et al.* [127]	Linear detection range 0.01–200 ng/mL. LOD 0.11 pg/mL.
Au/Ag	Bimetallic core-shells. Anti-CEA SERS probes used with electrochemical luminescence	2013	Chen *et al.* [128]	Linear detection (using luminescence) 1000 to 0.2 ng/ml. LOD 5 pg/mL.

In the two papers shown here mouse monoclonal antibody, used to achieve specificity, was used together with (i) Au/Pt NPs in an electrochemical immunoassay study by Cao *et al.* [127] and (ii) anti-CEA-functionalized 4-mercaptobenzoic acid–labeled Au/Ag core-shell bimetallic NPs as a SERS probe by Chen *et al.* [128]. The authors of each paper highlighted the possibility of taking their developed methods forward in the first case Cao *et al.* [127] commented that the results obtained make the resulting sensors with their biometallic nanochained structures promising candidates for the next-generation of sandwich-type electrochemical immunoassays, while Chen *et al.* [128] noted after obtaining good agreement (with a conventional electrochemical luminescence method) from 26 colorectal cancer patients, that the approach has the feasibility and potential for development as a clinical tool for analysis of tumor markers in the blood.

The remaining target analytes and the bimetallic constructs used for their detection considered in this review are presented in Table 7.

**Table 7:** Bimetallic constructs used for sensing miscellaneous analytes.

Target Analyte	Metals	Construct/Support/Detection Method	Year	Authors	Detection Limits/Sensitivities
Long non-coding RNA	Pt/Pd	Bimetallic nanodendrites/nanoflower-like clusters on graphene oxide/Au/horseradish peroxidase.	2015	Liu *et al.* [129]	Linear range 1.00 × 10^−3^ to 1.00 × 10^3^ pM/mL. LOD 0.247 fM/mL.
Carbohydrate antigen 19-9.	Au/Pd	Core/shell bimetallic functionalized graphene nanocomposite immunosensor.	2015	Yang *et al.* [130]	Linear range 0.015 to 150 U·mL^−1^. LOD 0.006 U·mL^−1^.
Cancer cells (human leukaemia CCRF-CEM)	Cu/Au	Iodide-responsive NP-based colorimetric platform.	2015	Ye *et al.* [131]	Linear range 50 to 500 cells/mL. LOD 5 cells in 100 μL binding buffer.
Dopamine	Ag/Pt	Electrospun nanoporous carbon nanofibers decorated with bimetallic NPs.	2014	Huang *et al.* [132]	Linear range 10−500 μM. LOD 0.11 μM (S/N = 3).
Cardiac troponin I	Pt/Au	Electrochemiluminescence immunosensor using poly(L-histidine)-protected glucose dehydrogenase on NPs to generate *in situ* co-reactant.	2014	Xiao *et al.* [133]	Linear response from 0.010 ng·mL^−1^ to 10 ng·mL^−1^. LOD 3.3 pg·mL^−1^ (S/N = 3).
Human Tissue Polypeptide Antigen (hTPA)	Pd/Pt	Bimetallic construct as secondary Ab on graphene/GCE. Amperometric sandwich type immunosensor.	2014	Wang *et al.* [134]	Linear range 0.0050–15 ng·mL^−1^, LOD 1.2 pg·mL^−1^.
Cancer Antigen 125 (CA125)	ReAu	Bimetallic NPs. ReAuCA125Ab–CA125 immunocomplex.	2011	Cai *et al.* [135]	Linear range 0.1–240 mU/mL. LOD 0.02 mU/mL
Glutamate	Au/Pt	GlutaOx-[C_3_(OH)_2_mim][BF_4_]-Au/Pt-Nafion. Electrocatalytic detection.	2011	Yu *et al.* [136]	Linear range 0.5 µM to 20.0 µM. LOD 0.17 µM.
Lead	Au/Pd	3-D ordered macroporous BM electrode/SWCNTs. Amperometric detection of Pb^2+^ using methylene blue.	2014	Chen *at al*. [137]	Linear range 1 × 10^−17^–1 × 10^−4^ M. LOD (Pb^2+^) 1 × 10^−19^ M.
Mercury	Au/Pt	Inorganic-organic hybrid nanocomposite modified GCE. Electrochemical assay using anodic stripping voltammetry.	2010	Gong *et al.* [138]	Linear detection to 10 ppb. LOD 0.008 ppb.
Zearalenone	Pt/Co	GCE carrying immobilized nitrogen-doped graphene sheets with captured and bimetallic NPs. Non-enzymatic/mediator-free biosensor.	2013	Feng *et al.* [139]	Linear range 0.005–25 ng·mL^−^^1^. LOD 2.1 pg·mL^−^^1^.
Organo- phosphates	Au/Pt	Acetylcholineesterase/choline oxidase immobilized with glutaraldehyde on a modified GCE. Electrocatalytic detection at low potential for detection of H_2_O_2_.	2009	Upadhyay *et al.* [140]	Linear ranges for paraoxon ethyl, sarin, and aldicarb 150–200 nM, 40–50 nM, and 40–60 µM respectively at 30%–40% inhibition of acetylcholine esterase.
2-Butanone	Au/Ag	Bimetallic alloy/CNT. Assay based on CV.	2012	Zhang *et al.* [141]	Linear anodic peak current response (determined by CV) to 2-butanone from 0.01% to 0.075% (*v*/*v*).

### 2.7. Human Tissue Polypeptide Antigen, Cancer Antigen 125, Carbohydrate Antigen 19-9, Long Non-Coding RNA, Leukaemia CCRF-CEM Cells

The three target antigens, namely human tissue polypeptide antigen (hTPA), cancer antigen 125 (CA125), and carbohydrate antigen19-9 (CA19-9) all serve as markers for malignant disease. CA19-9, an important carbohydrate tumor marker, is elevated in a range of malignancies, including pancreatic, colorectal, gastric and hepatic carcinomas. The determination of serum CA19-9 levels therefore is important for the early clinical diagnosis, staging, and evaluation of patient recovery. Tissue polypeptide antigen (TPA) is a protein produced and released by proliferating cells that has recently attracted interest as an almost universal tumor marker as it is found in the majority of human malignant tumors [142,143,144]. While much remains to be discovered about its role and structure [145] CA-125, is a glycoprotein commonly used a tumor marker that is known to be frequently elevated in the blood of some patients with specific types of cancers, especially ovarian cancer [122,123,146]. Although the sensors used to identify these markers of malignant disease all used immune complexation techniques in which the bimetallic catalyst was used to facilitate the analysis of a secondary analyte generated by the binding of an epitope specific antibody, their choices of bimetallic metallic pairings are completely different, *i.e.*, Au/Pd (Yang *et al.* [130]), Pd/Pt (Wang *et al.* [134]) and Re/Au (Cai *et al.* [135]).

A sensor using Pt/Pd for long non-coding RNA, known to upregulated hepatocellular carcinoma (HCC), was made by Liu *et al.* [129] who used Pt/Pd bimetallic nanodendrites/nanoflower-like clusters on graphene oxide/Au/horseradish peroxidase to enhance the sensor’s catalytic efficiency and sensitivity for the detection of HCC. In a different approach that also used a bimetallic construct containing Au for the detection of malignant disease, Ye *et al.* [131] created an iodide-responsive NP-based colorimetric platform, in which an aptamer-functionalized Cu/Au NP probe was used to quantitatively connect an iodide-catalyzed colorimetric assay with target recognition molecules to create an ultra-sensitive cancer cell detection method for human leukaemia CCRF-CEM cells.

### 2.8. Cardiac Troponin I

Xiao *et al.*, 2014 [133] made an immunosensor sensor for cardiac troponin I (cTnI), which serves as a sensitive biomarker for monitoring acute myocardial infarction (reference cTnI levels are normally lower than 0.4 ng·mL^−1^). The sensor was based on electrochemiluminescent detection using poly(l-histidine)-protected glucose dehydrogenase on Pt/Au bimetallic NPs to generate an *in situ* co-reactant (NADH) that enhanced the electrochemiluminescence signal, thereby enabling the sensor to demonstrate a lower detection limit for cTnI of 3.3 pg·mL^−1^.

### 2.9. Glutamate and Dopamine

Glutamate and dopamine (DA) are both important excitatory neurotransmitters in the central nervous system that play many important roles in brain functioning; e.g., glutamate is known to be involved in responses to stress [147] and the neuroendocrine output of the pituitary gland [148,149,150], while dopamine modulates the activity of reward and pleasure centers, and helps to regulate movement and influence emotional responses. As glutamate dependent neuronal pathways in brain are known to be implicated in many neurological disorders, the ability to accurately monitor of glutamate levels would contribute significantly to our fundamental understanding of the role of glutamate in these disorders [151]. Yu *et al.* [136] in their paper focusing on a sensing system for glutamate used a hydroxyl functionalized room temperature ionic liquid (RTIL), [C_3_(OH)_2_mim][BF_4_], to form a novel H_2_O_2_ biosensor fabricated with [C_3_(OH)_2_mim][BF_4_] as the substrate with electrodeposited bimetallic Au/Pt NPs. When immobilized with glutamate oxidase (GlutaO*_x_*), the fabricated GlutaO*_x_*-[C_3_(OH)_2_mim][BF_4_]- Au/Pt-Nafion construct displayed, as noted by the authors a “sensitive and reproducible” response to glutamate at a potential of −200 mV.

Similarly, due to its importance as an excitatory catecholamine neurotransmitter, implicated in disorders such as addiction, Parkinson’s disease, and schizophrenia there is an urgency associated with finding a sensitive, selective, and reliable method for the direct detection of DA. Whereas the approach to glutamate sensing employed a Au/Pt bimetallic pairing the approach to dopamine sensing adopted by Huang *et al.* [132] used Pt in conjunction with Ag formed as NPs, adhered to electrospun nanoporous carbon nanofibers supported on a glassy carbon electrode for the selective detection of DA in the presence of commonly found confounding analytes including uric acid and ascorbic acid.

### 2.10. Trace Meal Detection (Lead and Mercury)

Both lead and mercury are generally recognized as toxic metals that induce neurological damage, giving rise to various diseases, especially in young children. Industrialization and the historic use of leaded fuels have led to the persistence of lead in the environment creating a global health problem [152]. Some pathological consequences thought to be related to lead toxicity include neurological disorders, kidney damage, hypertension, reproductive disorders and the potential for carcinogenic effects [153,154]. For this reason the ability to detect the presence of lead at trace levels is crucial for environmental monitoring.

Mercury, the only metal that is a liquid at room temperature, exists in two forms -organic and inorganic, both of which are potentially toxic. Mercury, together with many of its compounds, is now widely distributed in air, water, and soil [155]. Lipophilic compounds, such as dimethylmercury and methylmercury (responsible for Minamata disease) are able to cross biological membranes (e.g., the blood/brain barrier) to accumulate in various organs, potentially leading to genetic dysregulation [156], together with both chronic and acute poisoning affecting organs such as the urinary, central nervous, endocrine, and gastrointestinal systems [157,158].

Chen *at al*. [137] in a paper entitled: “Target-induced electronic switch for ultrasensitive detection of Pb^2+^ based on three dimensionally ordered macroporous Au/Pd bimetallic electrode” showed the coupling of signal amplification from a three dimensionally ordered macroporous (3DOM) Au–Pd bimetallic electrode on supporting SWCNTs labels with substrate DNAs serving as a recognition element. Electroactive methylene blue was used as a signal reporter to enhance the electrochemical signals. This work demonstrated that with the application of a Pb^2+^ dependent DNAzyme, the sensing system can be made to be highly selective. The authors commented that this work has promising potential for the on-site testing of Pb^2+^ in drinking water and serum sample analysis.

A sensor able to detect and quantify Hg^2^^+^ should have significant utility for health/environmental monitoring. Gong *et al.*, used a GCE modified with a three-dimensional porous network nano-architecture, in which homogenously distributed bimetallic Au/Pt NPs served as microelectrode ensembles in a matrix of interlaced organic nanofibers [138]. The resulting voltammetry sensor was tested with tap and river water. The sensor’s detection limit was found to be 0.008 ppb, *i.e.*, well below the guideline value from the World Health Organization (WHO). Interference from other heavy metal ions such as Cu(II), Cr(III), Co(II), Fe(II), Zn(II), and Mn(II) ions, know to commonly interfere in mercury analysis, was effectively inhibited.

### 2.11. Zearalenone

Zearalenone (ZEA) is a potent non-steroidal mycotoxin produced by some fungal species. ZEA is heat-stable and is found worldwide in a number of cereal crops as well as in bread [159]. Ingestion of this toxin can result in infertility, abortion or other breeding problems, especially in farm animals such as pigs [160,161]. Although the bulk of the data collected to date relates to livestock animals exposed to ZEA-containing feed, with little data being available that points to endocrine disruption by ZEA in humans, the presence of ZEA (with its near steroidal oestrogenic potential) in the human food-chain must remain a cause for concern [161,162]. Feng *et al.* developed a sensitive biosensor for ZEN detection using a GCE carrying a nanoporous bimetallic alloy as a secondary antibody label (Ab2) [139]. The biosensor was prepared by immobilizing nitrogen-doped graphene sheets (N-GS) with a captured primary antibody (Ab1) on the GCE. The analytes were bound to Ab1 for further capture by alloyed nanoporous Pt/Co acting as an antibody label.

### 2.12. Organophosphates

Despite being commonly used as industrial solvents and plasticizers, organophosphates (OPs) are generally recognized as being highly toxic, with devastating effects on wildlife (particularly bees) and humans. Low levels of exposure are thought to have a negative impact on the neurobehavioral development of fetuses and children [163,164,165].

Although the 1925 Geneva Protocol prohibits the use of chemical warfare agents, including OP nerve agents, some 23 countries are thought to have chemical weapon stockpiles [163]. Due to their extreme neurotoxicity, OPs have the potential to be used as devastating chemical-warfare/terrorism agents. The increasing need to protect both military and civilian personal from attack by chemical warfare agents (CWAs) will spur the development of biosensors able to detect such threats in both military and civilian settings.

The applications of bimetallic NPs for the detection and identification of OP CWAs at low concentrations has been reported by Upadhyay *et al.* [140], whose sensitive amperometric biosensor was based on the electrodeposition of Au/Pt bimetallic NPs on a 3-aminopropyltriethoxy silane modified GCE. The resulting device enabled the detection of paraoxon ethyl, aldicarb, and sarin. The biosensor used a two enzyme approach, on an electrode, with acetylcholineesterase/choline oxidase being cross-linked with glutaraldehyde. Acetylcholineesterase was used to selectively recognize and hydrolyze the acetylcholine with the resulting choline being oxidized by choline oxidase in the presence of oxygen resulting in the production of H_2_O_2_. The synergistic action of Au and Pt showed excellent electrocatalytic activity when using a low applied potential for the detection of H_2_O_2_.

### 2.13. 2-Butanone (a Volatile Indicator of Various Cancers)

*Helicobacter pylori* infection is associated with gastric cancer, the second most common cancer in the world. The bacterial infection gives rise to a characteristic metabolic signature composed of various ratios of biomarkers [166]. Also, in lung cancer [167] and liver cancer [168,169] the volatile end-products of aberrant metabolism also potentially offer convenient diagnostic opportunities. In all cases, early diagnosis increases the possibility of patient survival. Among the compounds contributing to the disease’s metabolic signature is 2-butanone. Thus, sensitive methods of detection for this and related species offer hope in the search for accurate, rapid and cost-effective diagnostics.

A relatively novel approach to constructing a bimetallic construct for a biosensor able to quantifiably determine the presence of 2-butanone was developed by Zhang *et al.* [141] and presented in a paper entitled “Chloroplasts-mediated biosynthesis of nanoscaled Au/Ag alloy for 2-butanone assay based on electrochemical sensor.” While the use of microorganisms is regarded as a clean and environmentally friendly, although labor intensive, approach to the generation of NPs, the use of chloroplasts, which can be conveniently isolated in large amounts without the need for tedious culture work, offers a means of simplifying the process. Their paper highlighted a ‘one-pot’ method for the synthesis of an Au/Ag alloy using cellular chloroplasts as both reducing agents and stabilizers. Spectroscopic (FTIR) results showed the chloroplast’s proteins to be bound to the Au/Ag alloy through their free amino groups. The bimetallic Au/Ag particles were found to have only one plasmon band, thereby indicating the formation of an alloy structure. The Au/Ag alloy was dispersed into MWNTs to form a nano-sensing film, which exhibited high electrocatalytic activity for 2-butanone oxidation at room temperature.

The authors speculated that the sensor’s excellent electronic catalytic characteristics may be attributable to synergistic electron transfer effects in the Au/Ag alloy and MWNTs and further commented that the sensor could be expected to provide a means to develop a breath sensor able to detect early stage cancers, especially early stage gastric cancer.

Although it is well established that the resulting properties of bimetallic catalysts benefit from metal-to-metal synergistic effects and possess characteristics that are significantly different from their monometallic counterparts, any unifying rational that suggests why a given bimetallic construct is optimal for any chosen analyte remains elusive [170]. Thus, comparing the bimetallic couples as an entirety shows that few, if any, ongoing trends regarding bimetallic pairings have emerged.

## 3. Synthesis of Bimetallic Nanoparticles

The generation of materials whose sought after physical and chemical properties are derived from co-operative or synergistic effects between two metals, using controlled synthesis to create bimetallic nanomaterials with various architectures, is a field of endeavor with considerable ongoing interest [170,171,172]. An excellent comprehensive review focused on the use of colloidal chemistry to generate bimetallic catalysts with sought after morphologies is given by Gu *et al.* [173].

**Figure 4:**
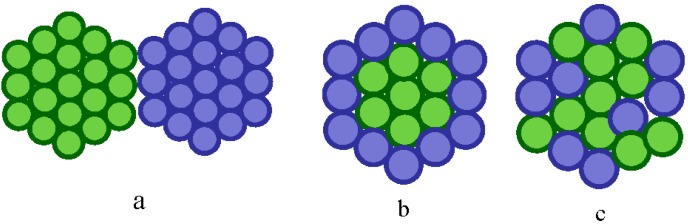
Catalytic structures: (**a**) heterogeneous, (**b**) core-shelled and (**c**) alloyed.

Based on the synthetic protocol employed, bimetallic NPs can be divided into three broad groups, herterostructured, core-shelled and alloyed, see Figure 4a–c respectively. Alloyed materials can be further subdivided into segregated, ordered and random homogeneous alloys. In heterostructured NPs, individual nucleation and growth of two kinds of metal atoms occurs with the sharing of a mixed interface during the growth process. Core–shell structures are formed if one type of metal is reduced first to form an inner core upon which acts as a support for another metal which may either grow by isolated nucleation or by the formation of a homogenous thin layer to form a shell. Alloy NPs are homogeneous mixtures of two metals at the atomic level and can be distinguished by the formation of metal-metal bonds.

The forces driving the mixing that occurs between two metals will determine the morphology of the final catalyst. Such forces include the relative strength of the bond between the two different metals compared to that which occurs between the two pure component metals. Higher overall alloy bond strengths, accompanied by charge transfer, promote intimate mixing; whereas lower interaction energies will not promote mixing leading to segregation of the two metals. Additionally, metals with higher surface energies, and those comprised of smaller atoms, are preferentially stabilized in the higher coordination environment of the material’s interior, thus promoting migration of the lower surface energy metal to the surface to form the shell [174].

### 3.1. Heterostructured Particles

In the wet-chemical synthesis of bimetallic nanocrystals, two metals nucleate and grow separately (heterogeneous seeded growth), due to their different standard reduction potentials. This can lead to the formation of heterostructured particles, Figure 4a. Seed-mediated growth is a common route to the formation of bimetallic nanocrystals, where the seeds of one metal serve first as the sites of nucleation and then for the growth for another metal [171,172,173,174,175,176,177]. The final morphology of the bimetallic structure will be determined by the type of growth exhibited by the second metal, e.g., layered, island or mixed [173]. The type of growth shown by the second metal is determined by several parameters, these include: matching/mismatching of lattice structures and their associated constants, surface and interface energy correlations, and the difference between the two metals electronegativities [178]. For two given metals, these variables direct seed growth towards either conformal or site-selective growth, thereby generating organized core–shells, with the second metal present as a thin-layer (see below), or other less organized structures [179]. Readers seeking a contemporary (2015) overview focused on the catalytic applications of heterogeneous novel metallic nanomaterials are referred to a recently published review by Wang and Gu [180].

#### 3.1.1. Core Shell Nanoparticles

Bimetallic core-shell nanomaterials, have an active-metal shell supported on another dimensionally-stable metal (commonly a noble or a 3d-transition metal) acting as a core. The core metal can be chosen not only to reduce active catalytic metal usage, but also to tailor the performance of the resulting NP [181,182]. The catalytic reaction usually occurs on the core-shell’s surface; thus, constructing the structure so as to have a thin catalytic-metal shell is a route to both enhancing activity and minimizing the cost of highly expensive metals such as Pt.

Bimetallic NP morphology is dependent upon the synthesis conditions. If in a simultaneous reduction process the standard reduction potentials of the two metals used is significantly different they will both nucleate and grow separately, thereby offering the possibility of generating core-shell structured particles, Figure 4b [172,176,182,183].

Bimetallic core-shelled NPs are commonly prepared by seed-mediated growth, or one pot co-reduction, of metallic precursors to allow the growth of a second metal on a seed resulting in the generation of a bimetallic structure with a controlled morphology and composition [181]. One-pot co-reduction resembles seed-mediated growth in so far as the two metal precursors are simultaneously added into the synthetic system, one metal ion cation will be the first to undergo reduction, due to the reduction potential difference between the two species. This first-reduced metal will provide the seeds for the reduction and growth of the second metal. When the heterogeneous nucleation and growth on pre-formed seeds of one metal follows a conformal path, the second metal will grow on all faces of the seed, leading to the formation of the core-shell structure [184]. Using this approach bimetallic core-shell nanocrystals with well-defined shapes, have been synthesized by many groups [171,185].

#### 3.1.2. Alloyed Structured Bimetallic Nanoparticles

Nanoalloys can be generated within a variety of media, such as colloidal solutions, on surfaces, or inside pores. Controlled chemical synthesis can be used to generate alloys with desired characteristics. By utilizing strong reducing agents which are capable of simultaneously reducing the metal precursors at optimized rates, alloyed structures can be fabricated [171,172,186]. Alternatively, by carefully adjusting the redox potentials of two metals; e.g., by employing selected surfactants, counter-ions, *etc.*, two different metal ions can be reduced at the same time to generate alloyed NPs.

Alloyed bimetallic NPs, as illustrated in Figure 4c, are formed by the intimate mixing of two metals. The use of a strong reducing agent is a means of achieving the co-reduction of two different metal ions that is commonly used to create alloy structured bimetallic NPs. Alloying Pt with suitable noble metals such Ru, Rh, Pd, Ir, Os, Ag, Au, or with non-noble 3d transition metals such as Cu, Fe, Co, or Ni, has been shown to be an effective route to the formation of electrocatalysts.

It has been reported that the catalytic activity and long-term stability of alloyed structures is dependent both on composition and structure [174,187]. Thus, the introduction of synergisms resulting from the complexity introduced by the degree of alloying in bimetallic NPs is critical in determining selectivity and catalyzed reaction rate [186].

To produce optimized alloyed bimetallic particles the reaction conditions must be controlled. Xu *et al.* [188] in approach to the design of bimetallic alloys, demonstrated a facile one-step room-temperature synthesis of Pt_3_Ni NP networks with improved electro-catalytic properties. The synthetic strategy they used to prepare bimetallic NPs (alloyed Pt_3_Ni) was an EG assisted two-phase synthetic method. The resulting Pt_3_Ni had improved catalytic activity with respect to the direct electrocatalytic oxidation of small organic molecules. The authors commented that their approach was applicable to the production of bimetallic alloys with adjustable composition.

## 4. Summary

Having, several decades ago, realized the importance of nanoscaled gold in promoting catalysis we have now made the transition from gold particles to bimetallic constructs. The introduction of a second metal into the catalyst affords opportunities to increase, through synergistic interactions resulting from alterations to both the geometric and electronic structures, both the activity and selectivity of the catalytic material.

Hybrid bimetallic nanostructures offer the possibility to combine metals, and optionally other components, in a myriad of combinations. Optimized synergistic metallic combinations, incorporated into sensors, now find application in devices for a host of applications. However, the fact that little, or more often no, rationale is offered in the papers for the disparate choice of metallic pairings, often for the same target analyte, other than the occasional reference to “facilitated electron transfer”, possibly indicates that there is little in the way of convergence in the approaches being adopted at the present time.

## 5. Conclusions and Future Prospects

The need for a review of bimetallic constructs used in catalysts testifies to the now generally accepted realization that binary nanostructured materials exhibit physical and chemical properties that are quite different from their individual components. Thus, the synergisms allowed by mixed 3d transition metal/metal oxides have been frequently shown to confer greater catalytic activity, which can be further enhanced and tailored for specificity.

The analysis of catalytic behavior, in related areas of endeavor (such as energy materials), has moved away from a narrow consideration of the local environment of the active site to a broader consideration of the complex dynamic interactions, which describe the interplay, between a range of structural and energetic parameters. However, due to the interaction complexity in systems comprising more than one metallic component the forces that control the metal–metal interactions occurring in bimetallic nanocatalysts remain poorly understood.

However, the cross-fertilization of approaches from related disciplines reliant on catalysts, especially in the field of energy production and utilization, may offer a route to the more structured investigation of suitable materials, thereby replacing the somewhat disparate bimetallic combinations with combinations derived from rationally targeted investigations. Some approaches to this are to be found in the application of X-ray absorption spectroscopy (XAS), using tunable X-ray beams, to determine the local geometric and/or electronic structure of the materials. Additionally, advances in *in silico* computational chemistry, for example the progress in density function theory (DFT) offer opportunities for the *ab initio* investigation of possible new materials free from the need for experimentally derived parameters. Such approaches should help to address the fact that while a host of bimetallic metal pairings have been presented, directed at an ever increasing range of target analytes, there remains in the literature a fundamental lack of understanding of the underlying processes; thus, improving our knowledge of the atomic level interactions of the combinations of metals used to make catalytic nanoparticles should enable us to fully realize their full potential.
